# CYP2C8 rs11572080 and CYP3A4 rs2740574 risk genotypes in paclitaxel-treated premenopausal breast cancer patients

**DOI:** 10.1038/s41598-024-58104-9

**Published:** 2024-04-04

**Authors:** Hanaa R. M. Attia, Mahmoud M. Kamel, Dina F. Ayoub, Shereen H. Abd El-Aziz, Mai M. Abdel Wahed, Safa N. Abd El-Fattah, Mahmoud A. Ablel-Monem, Thanaa M. Rabah, Amany Helal, Mona Hamed Ibrahim

**Affiliations:** 1 Medical Research and Clinical Studies Institute, Clinical and Chemical Pathology Department, Centre of Excellence, National Research Centre, Cairo, Egypt; 2 Clinical Pathology Department, National Cancer Institute, Cairo University, Kasr Al-Aini Street, From El-Khalig Square, Cairo, 11796 Egypt; 3 Medical Research and Clinical Studies Institute, Medical Biochemistry Department, Centre of Excellence, National Research Centre, Cairo, Egypt; 4 Medical Research and Clinical Studies Institute, Community Medicine Research Department, National Research Centre, Cairo, Egypt; 5 Baheya Centre of Early Detection and Treatment of Breast Cancer, Giza, Egypt; 6 Medical Oncology Department, National Cancer Institute, Cairo University, Cairo, Egypt

**Keywords:** Breast cancer, Cytochrome P450, Taxane, Menopausal status, Cancer, Molecular biology

## Abstract

Breast cancer (BC) is the most prevalent malignancy in women globally. At time of diagnosis, premenopausal BC is considered more aggressive and harder to treat than postmenopausal cases. Cytochrome P450 (CYP) enzymes are responsible for phase I of estrogen metabolism and thus, they are prominently involved in the pathogenesis of BC. Moreover, CYP subfamily 2C and 3A play a pivotal role in the metabolism of taxane anticancer agents. To understand genetic risk factors that may have a role in pre-menopausal BC we studied the genotypic variants of CYP2C8, rs11572080 and CYP3A4, rs2740574 in female BC patients on taxane-based therapy and their association with menopausal status. Our study comprised 105 female patients with histologically proven BC on paclitaxel-therapy. They were stratified into pre-menopausal (n = 52, 49.5%) and post-menopausal (n = 53, 50.5%) groups. Genotyping was done using TaqMan assays and employed on Quantstudio 12 K flex real-time platform. Significant increased frequencies of rs11572080 heterozygous CT genotype and variant T allele were established in pre-menopausal group compared to post-menopausal group (*p* = 0.023, 0.01, respectively). Moreover, logistic regression analysis revealed a significant association between rs11572080 CT genotype and premenopausal BC. However, regarding rs2740574, no significant differences in genotypes and allele frequencies between both groups were detected. We reported a significant association between CYP2C8 genotypic variants and premenopausal BC risk in Egyptian females. Further studies on larger sample sizes are still needed to evaluate its importance in early prediction of BC in young women and its effect on treatment outcome.

## Introduction

Breast cancer (BC) is the most common cancer among women in Egypt causing 22 percent of all cancer-related female deaths^1^. In 2018, it constituted 24% of new cancer cases and 15% of deaths worldwide^2^. It is a hormone dependent cancer carrying a great heterogeneity in the outcomes of patients with similar clinical features. It is important to investigate breast cancer in the context of menopausal status due to differences in causes, risk factors, molecular features, and disease outcomes^3^. Early detection of premenopausal (pre-M) breast cancer constitutes a great burden in low- and middle-income countries^3^.

When diagnosed it is more advanced and challenging to manage than post-menopausal (post-M) cancer breast^4^. One of its risk factors is the longtime exposure to high levels of estrogen through estrogen signaling pathway and via the toxic effects of highly reactive metabolic compounds^5,6^. Cytochrome P450 (CYP) enzymes which belong to monooxygenase are a large family of heme proteins involved in the biosynthesis and oxidative metabolism of sex hormones. Gene polymorphisms of CYPs have been vigorously implicated for the risk and prognosis of breast cancer^7–9^.

Furthermore, they have effects on treatment outcomes and drug metabolism. These effects range from lack of treatment efficacy to adverse toxic reactions. Meta-analysis of thirty-one studies on chemotherapy-induced peripheral neuropathy (CIPN) involving 4179 patients on various neurotoxic chemotherapeutic agents demonstrated that the prevalence of CIPN was 48%^10^. They related the adverse effects upon taxane-based therapy to genetic variables in CYP enzymes especially CYP2C8 and CYP3A4 with inconclusive findings^11^.

CYP3A4 is the most abundant Cytochrome P450 enzyme (30%) in adults and is expressed predominantly in the liver. It is responsible for oxidative metabolism of endogenous and exogenous hormones^12^. CYP2C8 is responsible for most of paclitaxel elimination and correlates with exposure to paclitaxel^13^.

To our knowledge there have been no previous studies on genetic variables of these CYP450 enzymes in pre-menopausal versus post-menopausal cancer breast. To understand genetic risk factors that may have a role in pre-menopausal breast cancer we investigated variables of the two cytochrome P450 enzymes (CYP2C8, rs11572080 and CYP3A4, rs2740574) in female cancer breast patients on taxane-based therapy and we evaluated their variation based on menopausal status.

## Patients and methods

In the current study one hundred and five female patients with histologically proven breast cancer have been enrolled from Baheya Centre for Early Detection and Breast Cancer Treatment between 2020 and 2022. All the assessed patients were diagnosed based on morphologic examination of the tumor tissues. Biopsy for histopathologic diagnosis and to perform hormonal receptors (ER, PR and HER2) was done for every patient. All participants were treated with neoadjuvant or adjuvant taxane-based chemotherapy (paclitaxel) as a single agent or combination therapy. Chemotherapy induced peripheral neuropathy (CIPN) was identified based on clinical and laboratory findings. All participants were informed about the study and its objectives before blood sampling. The study has been approved by both the Ethical Committee of the National Research Centre (no, 17-109) and Baheya-Research Ethics Committee (no.0317) in accordance with the ethical standards of the Declaration of Helsinki and written informed consents were obtained from all the patients.

All patients have been subjected to full history, clinical examination, and metastatic workup, including chest radiograph, abdominal sonar, and bone scan. Laboratory examination including CBC and biochemical analyses including ALT, AST, urea, and creatinine were sequentially assessed for cancer breast patients within 48 h before chemotherapy.

Inclusion criteria included age (≥ 18 years), performance status less than 3 in accordance with the Eastern Cooperative Oncology Group criteria (ECOG)^14^. Patients with comorbid disease conditions like severe liver disease or renal failure prior to treatment, peripheral neuropathy or vascular complications from hypothyroidism, hypercholesterolemia, hypertension or diabetes, varicella zoster, peripheral vascular disease, and autoimmune disease with vasculitis were excluded. These conditions are known to be associated with the development of peripheral neuropathy^15^.

### Blood sampling

Peripheral blood samples (10 mL) were withdrawn from all participants under complete aseptic conditions into plain and EDTA-containing vacutainer tubes for biochemical analysis, complete blood count and genomic DNA extraction.

### Genotyping of rs11572080 and rs2740574

Genomic DNA was isolated from the whole blood by the QIA amp DNA blood mini kit (Qiagen, Germany) in accordance with the supplier’s instructions using Qia Cube® automated nucleic acid extractor (Qiagen, Germany). A Nano Drop spectrophotometer (Nano Drop Technologies Inc., DE, USA) was used for measuring DNA concentration and purity.DNA was adjusted at A260/280 ratio between 1.7 and 1.9 and normalized to the recommended working concentration at ~ 25 ng/μL.Then DNA yield was stored frozen at − 20 °C for all recruited samples until further use. Genotyping and allele frequencies using TaqMan assays from Thermo Fisher Scientific, Catalog number: 4362691wereemployed on Quant studio 12 K flex real-time PCR system; CYP2C8 gene, C_25625794_10, rs11572080 (CYP2C8*3, c.416G > A > CYP2C8*3, g.2130G > A); CYP3A4 gene, C_1837671_50, rs2740574 (CYP3A4*15B, g.-392A > G > CYP3A4*1B, g.-392A > G)^16^.

#### Sequence of primers

RS 11572080 Context Sequence [VIC/FAM]

CTCTTGAACACGGTCCTCAATGCTC [C/T]

TCTTCCCCATCCCAAAATTCCGCAA

RS2740574 Context Sequence [VIC/FAM]

TAAAATCTATTAAATCGCCTCTCTC [C/T]

TGCCCTTGTCTCTATGGCTGTCCTC

The Taqman probe principle relies on the 5′–3′ exonuclease activity of Taq polymerase to cleave a dual- labeled probe during hybridization to the complementary target sequence and fluorophore-based detection. The amplification condition consists of an initial 2 min at 50 °C for optimizing the UNG enzyme, 10 min denaturation at 95 °C followed by 40 cycles of 30 s of denaturation at 95 °C, 30 s of annealing at 52 °C and 60 s of extension at 65 °C. Data analysis of the genotyping results and allele frequencieswere carried out by TaqMan Genotyper software. Software tools enable converting the raw data into genotype calls (homozygotes and heterozygotes).

### Statistical methods

All test data was converted and manipulated by using SPSS software program version 20.0. Data was analyzed, mean and standard deviation or standard error of mean and range were calculated for the quantitative data as age, tumor size, biochemical laboratory results. All categorical variables were summarized using frequencies and percentages. Comparison among studied cancer breast patients’ groups based on menopausal status was done by using the chi‐square test for categorical variables and using Student's *t*‐test for continuous data. P value was established to determine the statistically significant difference between them. The difference between groups were considered statistically significant when *p* < 0.05 and considered highly statistically significant when *p* < 0.01. Genotype frequencies found among all studied patients were compared with their expected frequencies under Hardy–Weinberg equilibrium using a χ^2^ test (*P* > 0.05). Logistic regression analysis was applied to test for association between rs11572080 CT genotype and premenopausal breast cancer expressed as categorical variables and odds ratios (OR) with 95% confidence.

### Ethical approval

The study has been approved by both the Ethical Committee of the National Research Centre (no, 17-109) and Baheya-Research Ethics Committee (no.0317) in accordance with the ethical standards of the Declaration of Helsinki.

### Informed consent

Informed written consent was obtained from all participants after the study objectives were explained and before blood sampling. Confidentiality of patient data was guaranteed.

## Results

Patients’ age ranged between 27 and 73 years old. Patients were stratified according to their menopausal status into pre-menopausal (n = 52, 49.5%) and post-menopausal (n = 53, 50.5%) groups. PTX was administered on a weekly basis in a dose of 80 mg/m2 IV over 3 h as an adjuvant in 41%, neo-adjuvant in 48.5% or palliative in 4.7% of the studied patients. Six patients received hormonal therapy, some patients got PTX with other chemotherapies e.g. Epirubicin–Cyclophosphamide (EC), (n = 35, 33.3%), or doxorubicin hydrochloride (Adriamycin) and cyclophosphamide (AC), (n = 48, 45.7%).

**Figure 1:**
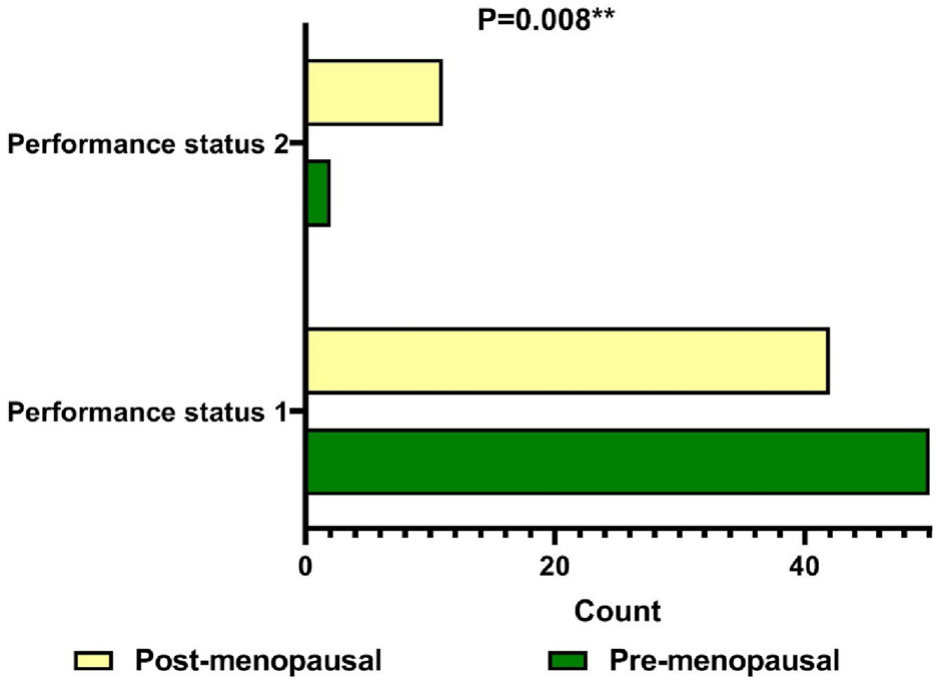
Peripheral neuropathy in the studied groups based on menopausal status.

**Figure 2:**
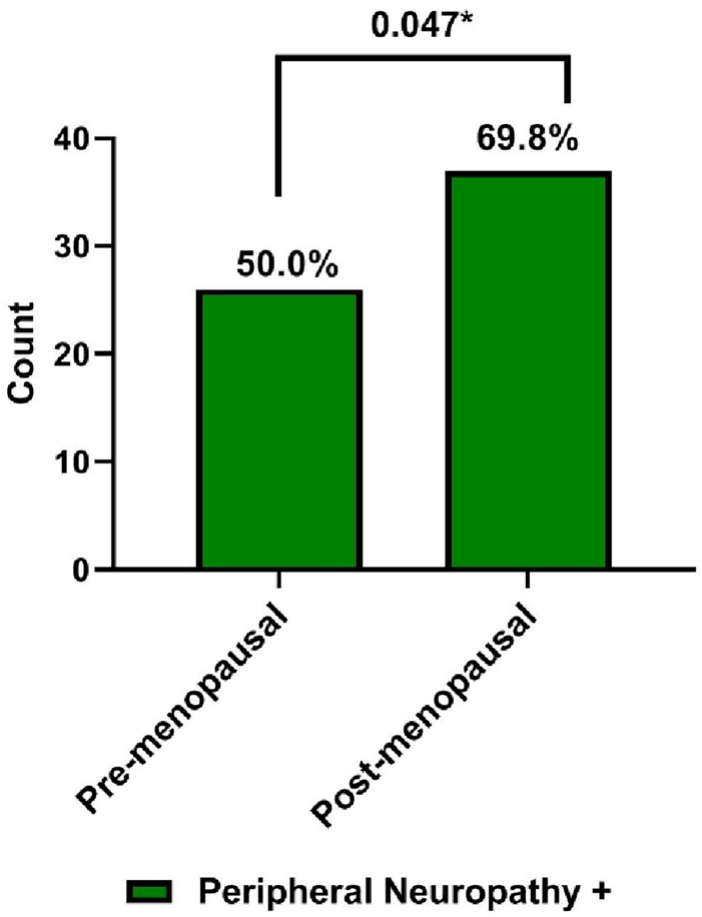
Performance status in the studied groups based on menopausal status.

**Figure 3:**
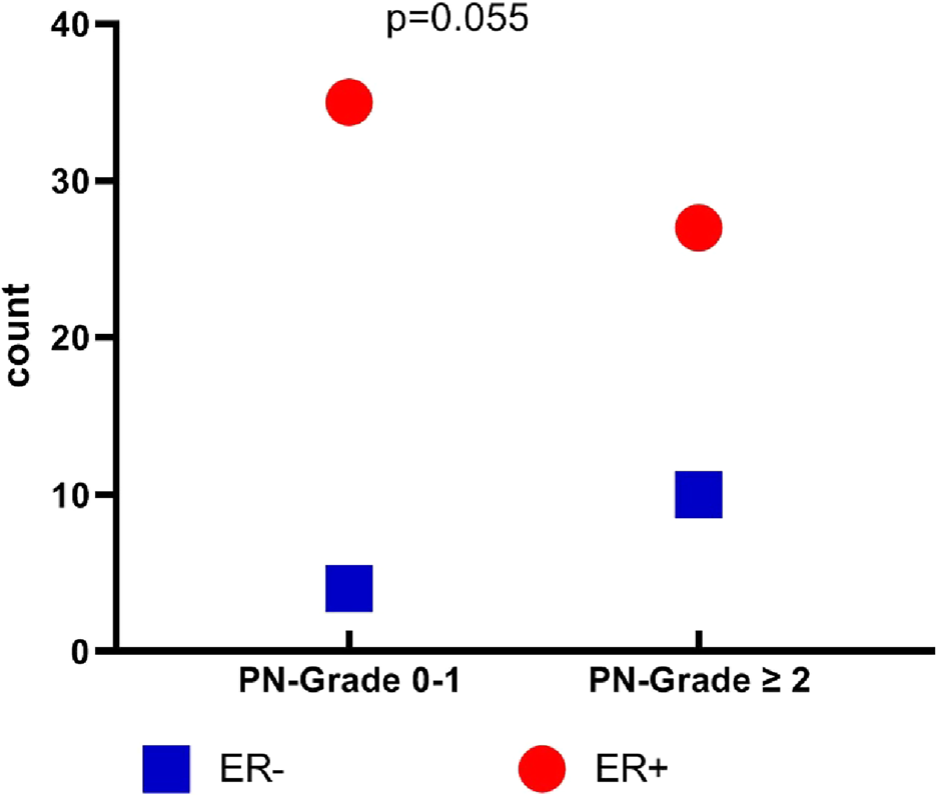
Estrogen receptor frequency in the studied groups based on peripheral neuropathy grades.

Clinical and laboratory findings in both groups are presented in Table 1. No statistically significant differences were found in tumor characteristics, pathology type and hormonal receptors’ frequencies between both groups. Performance grade 2 was significantly associated with post-menopausal breast cancer; as shown in Fig. 1; and with presence of Taxane-based CIPN (*p* = 0.008 and 0.01 respectively).CIPN was significantly more encountered in post-menopausal breast cancer group than the pre-menopausal patients (69.8%, *p* = 0.047, Fig. 2). High grade CIPN (grade ≥ 2) association with ER (estrogen receptor) negative cases was near significance, *p* = 0.055 as shown in Fig. 3.

**Table 1:** Clinical and laboratory findings in both pre- and post-menopausal patient groups.

Variable	Pre-menopausal N = 52 N (%)	Post-menopausal N = 53 N (%)	Odds ratio (95% CI)	*P* value
Age (y), mean ± SD	45.52 ± 7.19	60.43 ± 5.9	**–**	< 0.001**
Positive family history	14 (26.9)	12 (22.6)	**–**	0.603
Pathology type				
IDC	49 (94.2)	46 (86.8)	**–**	0.071
ILC	0	5 (9.4)		
Others	3 (5.8)	2 (3.8)		
Tumor side				
Right	21 (40.4)	21 (39.6)	**–**	0.932
Left	31 (59.6)	32 (60.4)		
Tumor size				
< 4 cm	27 (51.9)	22 (41.5)		0.363
> 4 cm	25 (48.1)	31 (58.5)		
ER+	41 (78.8)	40 (75.5)	**–**	0.368
PR+	43 (82.7)	46 (86.8)	**–**	0.589
HER2−	46 (88.5)	52 (98.1)	**–**	0.287
Pathology grade				
1	3 (5.8)	3 (5.7)	**–**	0.343
2	41 (78.8)	36 (67.9)		
3	8 (15.4)	14 (26.4)		
Clinical stage				
T (0–2)	22 (42.3)	17 (32.1)		0.493
(3–4)	30 (57.7)	36 (67.9)	**–**	
N (0–1)	37 (71.2)	34 (64.2)		0.704
(2–3)	15 (28.8)	19 (35.8)		
M (0)	46 (88.5)	46 (86.8)		0.537
− 1	6 (11.5)	7 (13.2)		
Performance status				
1	50 (96.2)	42 (79.2)	**1.944 (1.375–2.751)**	0.008*
2	2 (3.8)	11 (20.8)		
No. of cycles of treatment Mean ± SD	16.8 ± 10.3	17.0 ± 12.0		0.37
Peripheral Neuropathy	26 (50.0)	37 (69.8)	**2.273 (1.002–5.154)**	0.047*
Diarrhea	8 (16.7%)	12 (24.5%)	–	0.341
Gastritis	9 (18.8%)	8 (16.3%)	–	0.754
Fatigue	9 (18.8%)	11 (22.4%)	–	0.653
Skin rash	2 (4.2%)	1 (1.9%)	–	0.545
Nail Damage	2 (4.2%)	0	–	0.149
Nausea	10 (20.8%)	7 (14.3%)	–	0.396
Vomiting	10 (20.8%)	5 (10.2%)	–	0.148
Stomatitis	1 (2.1%)	0	–	0.309
Dyspnea	4 (8.3%)	3 (6.1%)	–	0.674
Bony pain	27 (52%)	26 (48%)	–	0.752

*P < 0.05 is considered significant; **p < 0.001is highly significant.

*N* number, *CI* confidence interval, *IDC* invasive ductal carcinoma, *ILC* invasive lobular carcinoma, *T* from 0 to 4 (higher T numbers indicates a larger tumor and/or spread to tissues near the breast), *N* from 0 to 3 (higher N numbers indicates that the cancer has spread to lymph nodes *M* 0 or 1 indicates if the cancer has spread to distant organs e.g. liver, lungs, or bones, *PR* progesterone receptor, *ER* estrogen receptor, *HER2* human epidermal growth factor receptor 2, *SD* standard deviation.

Significant values are in [bold].

**Figure 4:**
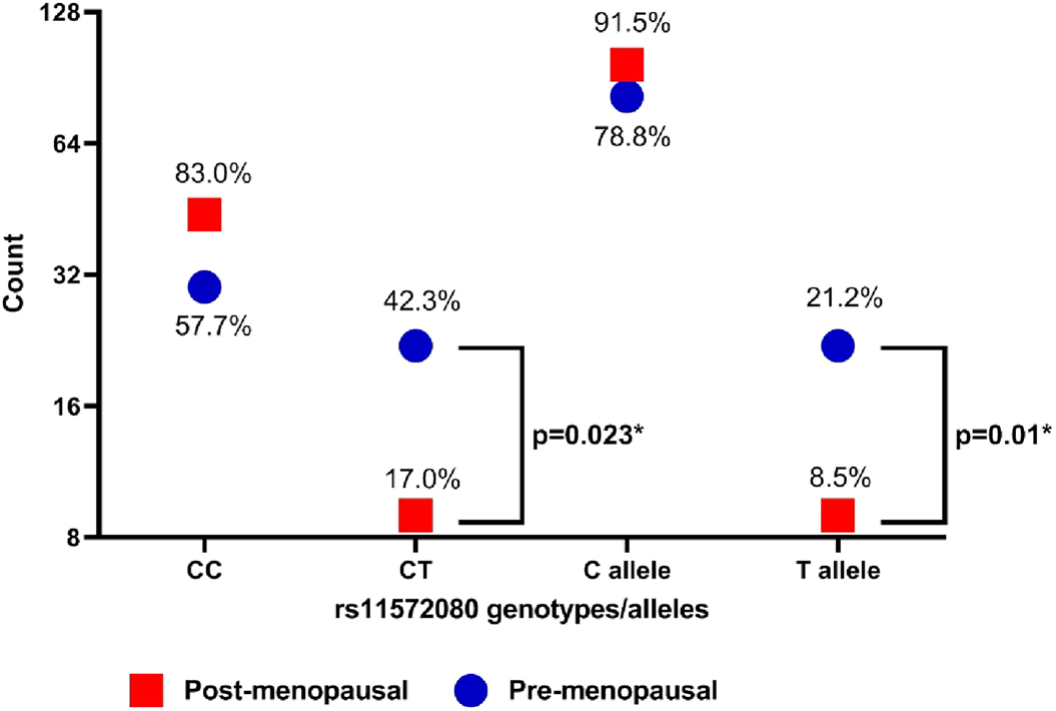
Frequencies of rs11572080 genotypes and alleles in the studied groups based on menopausal status.

Biochemical analysis results are shown in Table 2 with statistically higher urea and creatinine levels in the post-menopausal group. Genotypes and allele frequencies of all the studied patients are shown in Table 3. Genotypes’ frequencies are complying with Hardy–Weinberg equilibrium (*p* > 0.05). Increased frequency of rs11572080 heterozygous CT genotype was a significant characteristic finding in pre-menopausal breast cancer group (42.3%, *p* = 0.023) with variant T allele frequency of 21.2% vs. 8.5% in post-menopausal group as demonstrated in Table 4 and Fig. 4, (*p* = 0.01). Logistic regression analysis revealed a significant association between rs11572080 CT genotype and premenopausal breast cancer, *p* = 0.023.

**Table 2:** Biochemical findings in the studied patients (n = 105).

Variable	Pre-menopausal N = 52 Mean ± SD	Post-menopausal N = 53 Mean ± SD	*P* value
HB; g/dL	12.2 ± 1.27	12.5 ± 1.07	0.172
TLC; × 10^3^/µL	6.8 ± 2.1	6.9 ± 1.8	0.943
PLT; × 10^3^/µL	296.0 ± 64.4	287.2 ± 69.8	0.521
Urea; mg/dL	23.2 ± 7.1	31.2 ± 11.1	< 0.001**
Creatinine; mg/dL	0.76 ± 0.13	0.84 ± 0.18	0.025*
AST; U/l	20.8 ± 10.1	22.0 ± 10.3	0.570
ALT; U/L	21.1 ± 14.3	20.5 ± 11.4	0.819

*P < 0.05 is considered significant; **p < 0.001 is highly significant.

*Hb* hemoglobin, *TLC* total leukocytic count, *PLT* platelet count, *AST* aspartate transaminase, *ALT* alanine transaminase, *SD* standard deviation.

Significant values are in [bold].

**Table 3:** Frequencies of studied Genes among studied patients (n = 105).

Gene	Call rate (%)	Genotype/Allele	No. (%)	MAF, Global (1000genomes/our cohort	H.W.E
					P value
RS 2740574	100	CC	4 (3.8)	–	0.202
		CT	18 (17.1)		
		TT	83 (79.0)		
		**C**	26 (12.38)	**C** = 0.2308/0.1238	
		T	184 (87.6)	T = 0.7692/0.8762	
RS 11572080	95.8	CC	73 (69.5)		0.352
		CT	32 (30.5)		
		C	178 (84.7)	C = 0.9543 /0.8476	
		**T**	32 (15.2)	**T** = 0.0457/0.1524	

*MAF* minor allele frequency; risk allele is presented in bold; *H.W.E.* Hardy–Weinberg equilibrium.

**Table 4:** Frequencies of studied Genes among patients’ groups based on menopausal status.

Gene	Pre-menopausal N = 52 N (%)	Post-menopausal N = 53 N (%)	Odds ratio (95% CI)	*P* value
rs11572080 genotypes/alleles				
CC	30 (57.7)	44 (83.0)		
CT	22 (42.3)	9 (17.0)	**2.199 (1.071–4.513)**	0.023*
TT	0	0		
C allele	82 (78.8)	97 (91.5)		
**T** allele	22 (21.2)	9 (8.5)	**2.491 (1.204–5.154)**	0.01*
rs2740574 genotypes				
TT	40 (76.9)	42 (79.2)	**–**	
CT	9 (17.3)	8 (15.1)		
CC	3 (5.8)	3 (5.7)		0.961
T allele	89 (85.6)	92 (88.5)		
**C** allele	15 (14.4)	14 (13.5)		

Risk allele is presented in bold; *CI* confidence interval.

* < 0.05 is considered significant.

## Discussion

Breast cancer is the most diagnosed malignancy in women and the most common cancer overall, and its health and economic burden has been rising over the past decades in many parts of the world^17^. Breast cancer in pre-M women is frequently associated with worse prognosis compared to post-M women as it is more often diagnosed at a later stage of the disease^18^. Taxane-based chemotherapy regimens (e.g., paclitaxel and docetaxel) have been used as the first line of treatment in early-stage^19^. A frequent side effect is chemotherapy induced peripheral neuropathy CIPN, that occur in up to 70% of all treated patients and impacts the quality of life during and after treatment^20^.

Some factors such as increased dosage and age, are known to be associated with increased susceptibility of developing CIPN^21^. Moreover, there is a large interindividual variability independent of known risk factors suggesting that there could be an underlying genetic basis for susceptibility. Some single-nucleotide polymorphisms (SNPs) and other genetic variants may aid in predicting individual predisposition^22^. In the current study we aimed to verify genotypic variations in two cytochrome P450 enzymes involved in Paclitaxel metabolism in breast cancer patients and to identify their association with menopausal status.

In our cohort study, performance grade 2 and CIPN were significantly more prevalent in postmenopausal cancer breast patients than the premenopausal group. Hormonal fluctuations could be the reason for the adverse effects observed in post-menopausal cancer breast patients. The lower circulating progesterone level detected in such patients was one of the suggested mechanisms by Akshita et al.^23^ and was also confirmed by Sing and Su who have reported that progesterone exerts a neuro-protective effect through both genomic and non-genomic pathways^24^.

Progesterone has been authenticated as a neuroprotective hormone with beneficial effects on both central and peripheral nervous systems comprising promoting myelination, myelin repair and improving injuries of spinal cord and brain. Moreover, in an experimental study by Roglio et al.^25^ the use of progesterone reduced docetaxel-induced peripheral neuropathy in rats and prevented adverse changes in nerve conduction and consequently, it was considered as neuroprotective steroid in peripheral nerves^26^. Similarly, Ekici and Balkaya demonstrated that the protective effect of progesterone in rat model^27^. Prabhu et al.^28^ raised attention to a neuroprotective effect of premenopausal status, possibly related to higher circulating levels of progesterone. They hypothesized that progesterone administration prior to taxane-chemotherapy might protect against CIPN.

Blood estrogen levels dramatically decrease through peri menopause and further decrease for several years after menopause. It is likely that estrogen plays a role in neuroprotection or prevention of excessive neuronal excitability, thus, the decreased estrogen levels may be associated with the accelerated development of peripheral neuropathy. A clinical retrospective study by Miyamoto et al.^29^ showed that postmenopausal estrogen decline in female BC patients was considered a risk factor for therapy related peripheral neuropathy, and such a high-risk patient group, particularly, might require pharmacological intervention, except if the anti-cancer effect of paclitaxel is interfered. This was further supported by their preclinical study showing that ovariectomy in mice induced a somatic and visceral hyperalgesic state that could be reversed by estrogen**.**

Other mechanisms previously encountered in CIPN were explained by Starobova et al., including immune-mediated processes as loss of peripheral fibers, demyelination, axon degeneration, altered retrograde and anterograde transport, and mitochondrial dysfunction^30^. Increased incidence of CIPN with increasing age was demonstrated by Goreishi et al. who reported that advanced age is a significant risk factor for incidence and severity of neurotoxicity induced by chemotherapeutic agents including paclitaxel in particular^26^. In addition, Luclie et al.^31^ has stated that patients treated with paclitaxel appeared to be more at risk of developing persistent clinically significant CIPN especially if they were older than 75 and other potential factors were insignificant as regards being an ER positive or negative breast tumor.

Although genomic and molecular alterations play a significant role in breast cancer biology, studies that address the unique molecular changes in pre-M and post-M are limited^32^. Some studies have targeted differences in gene expression between pre-M and post-M breast cancer which were found exclusively in ER + breast cancer and suggested that the majority of differences was driven by altered hormonal levels^33^. Anders et al. analyzed microarray data from 784 early-stage breast cancers to discover gene sets able to distinguish breast tumors arising in younger women from tumors of older women^34^.

Prior studies have investigated somatic mutation analysis identifying 5 genes (CDH1, GATA3, MLL3, GPS2, and PI3KCA) for which mutation rates were significantly different between pre-M and post-M patients, where the overall mutation rates were lower in pre-M than post-M cases and that was likely a general effect of oxidative damage during aging rather than endocrine response^35,36^.

In our study, genotypes, and allele frequencies of all the studied patients showed significant increased frequency of rs11572080 heterozygous CT genotype in pre-menopausal breast cancer group with variant T allele frequency of 21.2 vs. 8.2% in post-menopausal group. Moreover, logistic regression analysis revealed a significant association between rs11572080 CT genotype and premenopausal breast cancer. In our opinion, this is a very interesting and promising finding as it reveals the association of this genotyping variation in early breast cancer patients and its importance in early prediction of cancer breast in young women.

## Limitation

The study limitation was that the relatively small sample size (150 BC patients) due to limited budget.

## Conclusions

CYP2C8 genotypic variants were significantly associated with premenopausal BC risk among Egyptian females. Larger scale genetic studies including large number of participants are still needed to elucidate the role of CYP2C8 gene polymorphism in the development and early prediction of BC in young women, in addition to its effect on treatment outcome.

It is very important to study attributable molecular risk factors that increase the chance of acquiring Breast cancer in young females, thus establishing prediction and screening programs which aims to offer appropriate drug regimens with improved patients' life outcome and emotional wellbeing.

## Data Availability

All data and materials are available and can be submitted when needed, Corresponding Author is responsible person who should be contacted if someone wants to request the data from this study.

## Abbreviations

BC: Breast cancer
pre-M: Premenopausal
CYP: Cytochrome P450
CIPN: Chemotherapy-induced peripheral neuropathy
ER: Estrogen receptor
SNPs: Some single-nucleotide polymorphisms
PTX: Paclitaxel
PN: Peripheral neuropathy
ECOG: Eastern Cooperative Oncology Group criteria
EC: Epirubicin-cyclophosphamide
AC: Adriamycin and cyclophosphamide
CIPN: Chemotherapy induced peripheral neuropathy
